# Minimum requirements for ookinete to oocyst transformation in *Plasmodium*

**DOI:** 10.1016/j.ijpara.2007.03.005

**Published:** 2007-09

**Authors:** Victoria Carter, Adéla M.L. Nacer, Ann Underhill, Robert E. Sinden, Hilary Hurd

**Affiliations:** a Centre for Applied Entomology and Parasitology, Institute for Science and Technology in Medicine, Huxley Building, Keele University, Staffordshire ST5 5BG, UK; b Infection and Immunity Section, Department of Biological Sciences, Sir Alexander Fleming Building, Imperial College of Science, Technology and Medicine, Imperial College Road, London SW7 2AZ, UK

**Keywords:** *Plasmodium berghei*, Ookinete, Oocyst, Basal lamina, Laminin, In vitro, Transformation

## Abstract

During their passage through a mosquito vector, malaria parasites undergo several developmental transformations including that from a motile zygote, the ookinete, to a sessile oocyst that develops beneath the basal lamina of the midgut epithelium. This transformation process is poorly understood and the oocyst is the least studied of all the stages in the malaria life cycle. We have used an in vitro culture system to monitor morphological features associated with transformation of *Plasmodium berghei* ookinetes and the role of basal lamina components in this process. We also describe the minimal requirements for transformation and early oocyst development. A defined sequence of events begins with the break-up of the inner surface membrane, specifically along the convex side of the ookinete, where a protrusion occurs. A distinct form, the transforming ookinete or took, has been identified in vitro and also observed in vivo. Contrary to previous suggestions, we have shown that no basal lamina components are required to trigger ookinete to oocyst transformation in vitro. We have demonstrated that transformation does not occur spontaneously; it is initiated in the presence of bicarbonate added to PBS, but it is not mediated by changes in pH alone. Transformation is a two-step process that is not completed unless a range of nutrients are also present. A minimal medium is defined which supports transformation and oocyst growth from 7.8 to 11.4 μm by day 5 with 84% viability. We conclude that ookinete transformation is mediated by bicarbonate and occurs in a similar manner to the differentiation of sporozoite to the hepatic stage.

## 1 Introduction

Malaria remains a major life-threatening disease in tropical and sub-tropical parts of the world, causing 300–500 million clinical cases, over one million deaths and severe economic and social losses (Sachs and Malaney, 2002). The causative agents of the disease, apicomplexans of the genus *Plasmodium*, are amongst the most studied parasites because of their devastating impact on human health and welfare.

*Plasmodium* parasites cycle between a vertebrate host and mosquito vector, experiencing changing environmental conditions as they develop from invasive to intracellular forms in the vertebrate, and invasive, intracellular and extracellular forms in the mosquito. Despite the importance of mosquito stages for malaria transmission, the majority of research on *Plasmodium* is focused upon the asexual stages that invade vertebrate erythrocytes; studies facilitated by the development, in 1976 (Trager and Jensen, 1976), of an in vitro culture technique that has rendered these stages more accessible to experimentation (Hurd et al., 2003).

In the mosquito, gametocytes complete their differentiation, fertilisation occurs and the zygote transforms into a motile ookinete within 24 h of having imbibed a blood meal. This extracellular ookinete migrates out of the blood bolus, traverses the midgut epithelium and transforms into a vegetative oocyst in the basal subepithelial space between the midgut epithelium and the basal lamina (BL). The young oocyst grows and undergoes sporogony, producing sporozoites that migrate to the salivary glands. Experimentation with systems that substitute for conditions in the vector for a period of some 50 years finally led to the development of methods to culture all the mosquito stages of several malaria species. Species that have been successfully cultured from gametocyte to sporozoite include *Plasmodium falciparum* (Warburg and Schneider, 1993), *Plasmodium gallinaceum* (Warburg and Miller, 1992), *Plasmodium berghei* (Al-Olayan et al., 2002a) and *Plasmodium yoelii* (Porter-Kelley et al., 2006). For one of these species, *P. berghei*, a method for the efficient production of ookinetes in vitro has been developed (Rosales-Ronquillo and Silverman, 1974; Weiss and Vanderberg, 1977) and this has provided material for detailed studies of gametogenesis, fertilisation and zygote development. Several studies have demonstrated that initiation of *Plasmodium* gametogenesis requires specific triggers. The change in environment from a mammalian to insect host provides cues such as a drop in temperature, and a mosquito factor identified as xanthurenic acid (Nijhout and Carter, 1978; Arai et al., 2001) which can regulate gametogenesis. These triggers, or substitutes for them, are present in the in vitro systems used to produce ookinetes (Weiss and Vanderberg, 1977; Carter et al., 2003).

The next critical event in the development of the sporogonic stages of *Plasmodium* is that of the transformation of ookinete to oocyst. Our knowledge of this phase of development is minimal and the process of rounding up of the ookinete to form an oocyst had not been studied in vitro previous to this work. We have identified components of the culture system that are essential requirements to initiate and sustain the formation of young oocysts from ookinetes.

It had been proposed that contact with components of the BL may act as a trigger for ookinete transformation in vivo (Weathersby, 1952; Meis and Ponnudurai, 1987; Ramasamy et al., 1997; Adini and Warburg, 1999; Schneider and Shahabuddin, 2000; Dessens et al., 2003; Arrighi et al., 2005; Vlachou et al., 2006) and culture systems that allow the transformation of ookinetes to oocysts, oocyst growth and sporozoite production have included a BL substitute, Matrigel and a co-cultured cell line such as *Drosophila melanogaster* S2, that may also contribute molecules present in the mosquito BL (Warburg and Miller, 1992; Warburg and Schneider, 1993; Al-Olayan et al., 2002a). Matrigel, an extracellular matrix derived from Engelbreth–Holm–Swarm mouse sarcoma, is comprised of laminin (56%), collagen IV (31%), enactin (8%), heparan sulfate proteoglycans and growth factors (BD Biosciences). Several ookinete proteins, such as the surface proteins P25/P28 (Siden-Kiamos et al., 2000), secreted ookinete adhesive protein (SOAP) (Dessens et al., 2003), circumsporozoite- and TRAP-related protein (CTRP) (Dessens et al., 1999) and von Willebrand factor A domain-related-protein (WARP) (Yuda et al., 2001), that are reportedly essential for ookinete and oocyst development in vivo, have been shown to bind to BL components such as laminin and collagen (Adini and Warburg, 1999; Arrighi et al., 2005). This binding could provide a signal that induces transformation, thus adding weight to the hypothesis that contact with the BL may be essential for parasite development (Dessens et al., 2003). Alternatively, other components of the culture system such as those present in the insect cell medium used as a substitute for haemolymph, may be essential requirements for ookinete transformation.

In vivo, ookinete transformation is a transient and unsynchronised event but in vitro we can observe the process occurring in thousands of ookinetes at any one time. Additionally, controlled interventions that enable careful dissection of putative transformation signals can be made and the minimal nutritional/environmental requirements for sporogonic development can be assessed. Using ookinetes that had been obtained in vitro (Carter et al., 2003) as a starting point, we have been able to describe the process of transformation and to identify a novel transient stage that we have called a transforming ookinete or took. We have demonstrated that, contrary to previous suggestions, BL components, and in particular laminin, are not required for transformation and early oocyst growth and that transformation appears to be a two-step process in which initiation is bicarbonate-dependent and complete transformation requires a range of nutrients.

## 2 Materials and methods

All reagents were purchased from Sigma unless otherwise specified.

### 2.1 Parasites and ookinete harvesting

Experiments were carried out in accordance with the UK Animals (Scientific Procedures) Act 1986 using approved protocols. CD mice were treated with phenylhydrazine 2 days prior to infection by i.p. inoculation with *P. berghei* (clone 259cl2 expressing green fluorescent protein (Franke-Fayard et al., 2004)) obtained from a donor mouse between the second and sixth passage from cryopreserved stock. Infected blood was collected from mice with a parasitaemia of 10–20% via cardiac puncture into a heparinised syringe. Gametocytaemic blood was passed through a pre-equilibrated, 5 ml sterile column containing 1 ml glass wool and 3 ml of Whatman CF11 cellulose powder (Beckton & Dickenson) to remove white blood cells. The blood was collected into T25 culture flasks with RPMI 1640 medium supplemented with 24 mM sodium bicarbonate, 0.4 mM hypoxanthine, 10,000 U penicillin/10 mg streptomycin and 20% FBS in a 1:10 ratio (blood: media). Gametocytes developed into ookinetes after 18 h of culture at 19 °C.

Ookinetes were separated on a MidiMacs (Miltenyi Biotec, Germany) separation system as previously described (Carter et al., 2003). Briefly this involved passing the blood and supplemented RPMI mixture through a pre-separation filter (to remove cellular debris and coagulated blood) and a MidiMac LS column mounted on a magnet. Ookinetes adhere to magnetised beads packed within the column and can be eluted by removing the column from the magnet. The rate of blood flow was reduced by adding a 23G needle to the base of the LS column to increase parasite contact time with the column.

### 2.2 Culture conditions

Culture conditions were as previously described (Al-Olayan et al., 2002a). Ookinetes were seeded at a density of 1 × 10^4^ per well into 8-well LabTek chamber slides or into 48-well plates (IWAKI) and cultured in oocyst culture medium (OCM: see Table 1) unless otherwise stated. Wells were either pre-coated with Matrigel diluted 1:1 with Schneider’s *Drosophila* insect medium or incubation took place in non-coated chamber slides. Parasites were either co-cultured in a 10:1 ratio with *D. melanogaster* S2 cells (Matrigel + S2), cultured in non-coated chamber slides with S2 cells (no-coating + S2), or in non-coated slides without co-cultured cells (no-coating − S2). Parasites were maintained at 19 °C for 5 days, unless otherwise stated. Media was replenished on day 3 (100 μl removed and replaced). All experiments were performed with six replicate wells on two or three separate occasions.

*Drosophila melanogaster* S2 cells were maintained in T25 culture flasks (Griener Bio-One) at 27 °C in supplemented Schneider’s insect cell culture medium (ICM) with 10% heat-inactivated insect cell culture tested FBS, penicillin (50 U/ml) and streptomycin (50 μg/ml).

### 2.3 Assessment of viability and transformation

Initially, a comparison was made between a Live/Dead viability/cytotoxicity kit (Molecular Probes) used according to the manufacturers’ instructions, and Erythrosin B (0.1% in PBS diluted 1:1 with the parasite culture medium) exclusion (Krause et al., 1984). No difference was detected between the results for cell viability between these tests (data not shown) and the latter test was used routinely in this study. Material was removed from culture wells and centrifuged at 2000*g* for 1 min to pellet parasites or insect cells. For parasite transformation rates, sample pellets were resuspended in 5 μl of PBS and 5 μl of Erythrosin B and mounted onto a glass slide for quantification. One-hundred live parasites were counted for each condition under oil immersion, and classified according to their morphological appearance of ookinete (banana), transforming ookinete (snail-like) or oocyst (sphere with a minimum diameter of 7 μm and highly motile internal contents). Counts were performed on triplicate samples. Transformation rates were determined by counting the number of live (Erythrosin B-excluding) ookinetes present on the initial day of culture, compared with the number after transformation (disappearance of ookinetes with concomitant increase in young oocysts denotes transformation as non-transforming ookinetes remain intact for several days).

For visualisation of nuclear material, ookinetes were fixed for 10 min in 1:1 ice cold acetone:methanol, washed and then incubated with anti-P28 monoclonal antibody for 1 h at room temperature before further washing and incubation with an anti-mouse IgG antibody diluted 1:200 for 1 h. Samples were washed again then mounted in Vectashield containing DAPI (Vector Laboratories) and visualised on an inverted Lieca DM IRB fluorescence microscope (Lieca Microsystems, Germany).

### 2.4 Electron microscopy

*Plasmodium berghei* samples were pelleted at 500*g* for 4 min 2 days post-culture and fixed in 2.5% glutaraldehyde in sodium cacodylate buffer (0.1 M, pH 7.4). Samples were post-fixed in 1% osmium tetroxide dispersed in 3% agar, dehydrated in graded ethyl alcohols and embedded in Spurr’s resin following standard protocols. Ultrathin sections, cut with a diamond knife on a Reichart “Ultracut E”, were mounted on grids and stained with uranyl acetate. Grids were examined with a JOEL 1230 TEM and images recorded using “analySIS” software.

### 2.5 Ookinete rescue

Ookinetes were observed to remain viable when cultured in PBS alone for up to 7 days, as determined by Erythrosin B exclusion, but did not transform. To initiate transformation, PBS (100, 200 or 300 μl) was removed from the culture well and replaced with 100, 200 or 300 μl of full-oocyst medium on day 1, 2 or 3 of culture. Samples were centrifuged at 2000*g* for 1 min and parasite viability was assessed as above.

### 2.6 Mosquito infections

*Anopheles stephensi* were starved for 24 h prior to bloodfeeding for 20 min on gametocytaemic CD mice infected with *P. berghei* (PbGFP_CON_ clone 259cl2). The mosquitoes were thereafter maintained on a 4% glucose solution and dissected in PBS 26–27 h p.i. Midguts from infected mosquitoes were cut open and the blood meal vigorously washed out with PBS before examination under oil emersion on the inverted fluorescence microscope for the presence of tooks on the BL side of the gut.

### 2.7 ELISA

*Drosophila* S2 cells have been reported to secrete laminin (Fessler et al., 1987). To confirm secretion of laminin by our cell line, *Drosophila* S2 cells from a confluent flask were counted on a haemocytometer and the total volume of supernatant from the culture was measured to calculate the number of cell equivalents per 100 μl of medium. Additionally, conditioned medium was collected from wells in which 1 × 10^5^ cells were incubated for 1, 3, 5, 7 or 9 days at 19 °C in 400 μl of OCM (2.5 × 10^4^ cells per 100 μl) to determine if laminin secretion could be detected in parasite culture conditions.

ELISA plates were coated with 100 μl of conditioned medium, diluted 1:50 in coating buffer (15 mM Na_2_CO_3_, 35 mM NaHCO_3_, pH 9.6) and serial dilutions made thereafter to 1:2000 and allowed to bind overnight at 4 °C. Plates were washed, blocked and incubated with antibodies raised against the β- and γ-chains of *Drosophila* laminin (Kumagai et al., 1997) diluted at 1:1000 in milk/PBS/Tween. Plates were then washed, incubated with an alkaline phosphatase (AP)-conjugated secondary antibody (Sigma) diluted 1:1000 in milk/PBS/Tween, and washed again. The substrate, *p*-nitrophenylphosphate, was used for visualisation. Control wells had primary antibody omitted. In addition, Schneider’s medium and FBS were tested for laminin and its absence confirmed.

### 2.8 Detection of laminin bound to culture plates

To establish whether laminin could bind to culture plates, making it unavailable in solution to developing parasites, culture plates were incubated for 24 h at 19 °C with 100 μg of mouse laminin in PBS. The plates were washed with PBS and assayed for laminin by immunofluorescence using a polyclonal anti-laminin antibody directed against all three chains. Similarly, *Drosophila* S2 cells were cultured in OCM for 24 h, the cells were then removed, and the culture plates assayed to determine whether laminin secreted by the cells was bound to the plate.

### 2.9 Minimal media

Using sterile PBS as a base, components of OCM (Schneider’s medium, heat-inactivated FBS, lipoprotein and cholesterol, NaHCO_3_ and hypoxanthine) were added singularly or in combination at appropriate concentrations. During further medium minimisation, Albumax II (Invitrogen) was used as a replacement for FBS and Schneider’s medium was replaced by individual components in groups of (i) amino acids, (ii) inorganic salts and (iii) other components, as listed by the manufacturer.

### 2.10 Media pH

Full-media were used for studies on the effects of the media pH. Media were adjusted by adding 5 M NaOH at room temperature before filtration (0.22 μm). However, as pH was found to steadily rise during the culture period, 25 mM *N*-2-hydroxyethylpiperazine-*N*′-2-ethanesulfonic acid (Hepes) was also used as a buffer to maintain initial pH values of 6, 7, 8 and 9. PBS adjusted to pH 6, 7, 8 or 9 was used for comparison.

### 2.11 Culture vessels

In the absence of either Matrigel or co-cultured cells, ookinetes were observed to adhere to the surface (IWAKI Cat. No. 3830-048); as determined by no movement upon gentle agitation. To eliminate possible cues received from adhesion, ookinetes were seeded into non-treated IWAKI multiwell plates (Cat. No. 1830-048) at the same density. To remove potential transformation cues received from mutual adhesion, ookinetes were placed one per well in treated and non-treated plates.

### 2.12 Statistical analyses

Data were analysed using Minitab version 14. The Anderson-Darling and Bartlett’s test were used to test for normal distribution and equality of variance, respectively. Data from repeat experiments were compared using a General Linear Model and, if not significantly different, pooled, tested for normality and equality of variance and analysed using the General Linear Model to compare transformation rates, growth and viability across conditions and across time.

## 3 Results

### 3.1 Markers for viability

A major constraint in characterising the process of transformation has been to demonstrate conclusively that an ookinete has undergone differentiation to become an oocyst. Using *P. berghei* (PbGFP_CON_ clone 259cl2) that constitutively expresses green fluorescent protein (GFP), we have observed that GFP expression, although becoming faint, often persists in parasites permeable to Erythrosin B. GFP expression was therefore deemed a less reliable viability indicator in our culture system.

Viability of ookinetes at the outset typically ranged from 80% to 90%. Parasites continued to exclude the vital dye Erythrosin B after rounding up to form oocysts, thus supporting the view that the changing morphology that we observe during transformation is not associated with dying ookinetes.

### 3.2 Markers for transformation

A *P. berghei* ookinete transforms from a banana-like shape, approximately 10 μm in length, to become a spherical young oocyst with a diameter of 7–8 μm. In an attempt to find mRNA markers that would help to identify a newly transformed oocyst and distinguish it from other parasite stages that may be present in the culture, we looked at the following well-characterised housekeeping or putative ookinete/early oocyst-specific molecules for signs of changes in transcription: CTRP, WARP, sporozoite microneme protein essential for cell traversal (SPECT), Myosin A, hypoxanthine–guanine phosphorybosyltransferase (HGPRT), scavenger receptor, P28, RNA S and circumsporozoite protein (CSP). However, none of these were suitable as no change in transcript abundance occurred during the transformation process or in the early oocyst (data not shown). We were therefore unable to positively identify the presence of early oocyst-specific features by PCR analysis and assessment has relied on morphological observations and the demonstration of growth.

### 3.3 Morphological and ultra-structural description of transformation

**Fig. 1:**
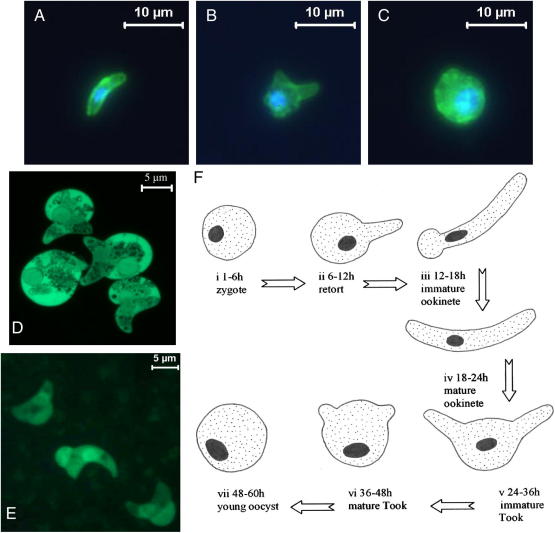
Formation of young *Plasmodium berghei* oocysts in vitro. (A–C) P28 (surface) and DAPI (nuclear) staining of ookinetes (A), tooks (B) and young oocysts (C). (D) PbGFP_CON_ tooks during late transformation in culture. (E) PbGFP_CON_ early transforming ookinetes in *Anopheles stephensi* midguts 27 h post-blood feeding (composite of three images). (F) Image adapted from Sinden et al. (1987) (i–iv) to incorporate ookinete to oocyst transformation (v–vii). Transformation begins with a small hump forming on the outer, convex, edge of the ookinete, often towards the apical end. Transforming ookinetes (tooks) then take on a snail-like appearance (v) and the nucleus is observed to move into the developing oocyst. Remnants of the ookinete tips are clearly visible (distinguishing these forms from zygotes; (vi) just prior to the formation of spherical young oocysts. The entire population of ookinetes transform in 12–36 h, depending on nutrient availability.

The in vitro transformation of *P. berghei* ookinetes to young oocysts follows a precise morphological pattern, distinct from that of zygote through retort to ookinete. Whereas immature ookinetes form via an extension from a spherical zygote, projecting from just one region (Sinden et al., 1985, 1987), fully mature, banana-shaped ookinetes progress to oocysts by rounding up via the formation of a protrusion in the middle of the ookinete that always originates on the outer convex edge. We have identified this phase as a transforming ookinete (took) (see Fig. 1A–C and F). The nucleus is in the hump, the two-tail ends of the ookinete are still visible in late tooks (Fig. 1B and D) and DAPI-positive material appears to increase in size when in the newly forming oocyst (Fig. 1C). We regarded fully rounded tooks as having completed transformation to an early oocyst. Within each culture, the process of transformation of mature ookinetes to oocysts is asynchronous but all viable ookinetes have become oocysts in 24–48 h. Ookinetes that have permeable membranes do not complete transformation.

**Fig. 2:**
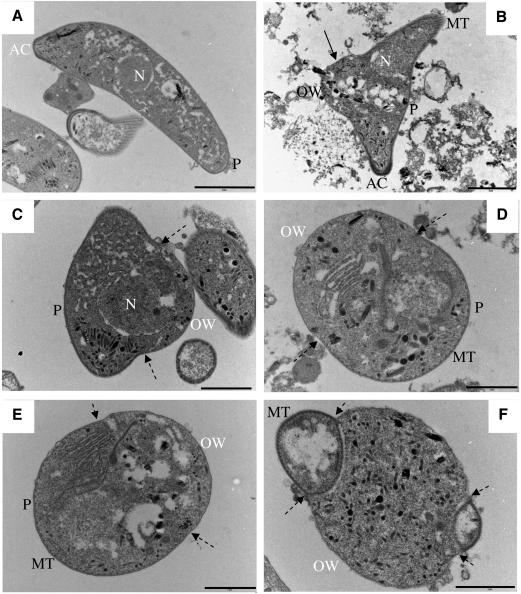
TEM images of an ookinete (A), and tooks (B–F) in vitro. (A) Ookinete, with nucleus (N), pellicle (P) and apical complex (AC) visible. (B) Early took: the AC is still clearly visible as are the microtubules (MT) and the beginning of the early oocyst wall, forming a hump on the outer ookinete edge. Membrane generated during this process differs from the ookinete pellicle (single arrow), and forms the basis of the oocyst wall (OW). (C) Early took: nuclear material can be seen to have moved into the newly formed area. Double and single membranes define the new OW and ookinete pellicle. Broken arrows indicate the transition areas from double to newly formed single membranes. (D and E) Mid-stage tooks showing microtubules below the remaining pellicle. (F) Late took: remnants of the ookinete tips with double membranes can be seen at either end of the forming oocyst: scale bar: A–C, 2 μm; D–F, 1 μm.

*Plasmodium berghei* (PbGFP_CON_) tooks have also been observed in vivo on mosquito midguts 28 h post-feeding on gametocytaemic blood (Fig. 1E). At the ultra-structural level, in vitro, the single plasmalemma of the newly forming oocyst can be distinguished from the triple membrane of the ookinete pellicle and microtubules are no longer visible in this area (Fig. 2B–F). A distinct junction between the ookinete pellicle and the single plasmalemma can be seen (Fig. 2D and E) and the nucleus can be observed in the hump of the took (Fig. 2C).

### 3.4 The role of basal lamina components and co-cultured cells in ookinete to oocyst transformation

Following incubation of gametocytaemic blood for 18 h, cultures were enriched for ookinetes; however, they still contained asexual stage parasites, gametocytes and zygotes in the process of transforming into ookinetes. The proportion of ookinetes in the total parasite population decreased from ∼25% on day 0 to ∼3% on day 1 and less than 1% on day 2 post-culture. No tooks were present on day 0 but they represented approximately 10% of the total fluorescing parasites when viewed on days 1 and 2, and a concomitant increase in diameter of spherical parasites from approximately 7 to 8 μm occurred from day 1 to day 2. As development can only proceed in one direction, these spheres were regarded as newly transformed oocysts. Over 90% of the ookinetes cultured with *Drosophila* S2 cells in the presence of the basal lamina substitute, Matrigel, transformed into young oocysts (termed maximal transformation rate) over a 1 to 2 day period and underwent some growth in diameter by day 3.

**Fig. 3:**
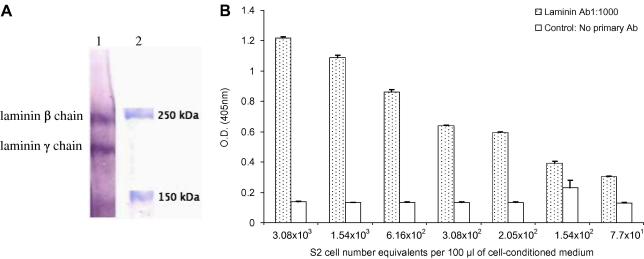
Production of laminin by *Drosophila melanogaster* S2 cells. Cells were cultured at 27 °C in Schneider’s Insect Cell Medium supplemented with FBS and Pen/Strep. (A) Western blot to visualise laminin secreted into the medium. The supernatant from a confluent flask was subjected to SDS–PAGE and transferred to a nitrocellulose membrane. The blots were probed with anti-*D. melanogaster* laminin-1 (β- and *γ*-chains) polyclonal antibodies (lane 1). Molecular markers are shown on the right (lane 2) and the corresponding molecular weight labelled. (B) Laminin secretion by S2 cells was determined by ELISA and readings are expressed as cell equivalents per 100 μl of medium. Error bars = SEM and *n* = 3 wells.

Two potential sources of BL components had been identified; namely Matrigel and the *Drosophila* S2 cells, which have been reported to produce basal lamina components such as laminin (Fessler et al., 1987). In our hands, S2 cells secreted the β- and γ-chains of laminin into supplemented Schneider’s medium (Fig. 3A) and laminin was detected at levels more than twofold above background from 200 cell equivalents from a confluent flask of *Drosophila* S2 cells (Fig. 3B), thus confirming that the cell line used can secrete laminin. However, when cells were cultured with OCM no laminin was detectable in the medium or bound to the culture plate (IFAT) on days 1–9 post-culture. Taken together these results suggest that laminin is not secreted in detectable amounts by the *Drosophila* S2 cells in our in vitro culture conditions.

**Fig. 4:**
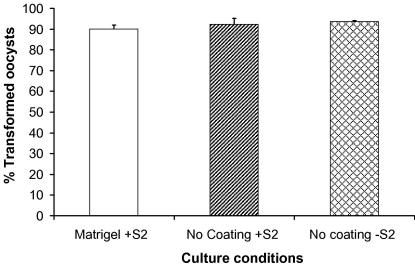
Maximal ookinete to oocyst transformation rates 24-h post-culture. Transformation rates were assessed by counting the number of parasites out of 100 that had changed from ookinete shape to young oocysts. No significant difference (ANOVA, *F*_(2,2)_ = 0.90, *P* = 0.422) was observed between full-culture conditions, culture without Matrigel and culture without Matrigel and *Drosophila* S2 cells. Bars represent SEM. The experiment was repeated three times and parasites counted in three separate wells for each condition.

Upon removal of Matrigel or Matrigel and *Drosophila* S2 cells from the cultures, no significant difference was detected in the percentage of transformed parasites between culture conditions (with and without cells or Matrigel) (ANOVA, *F*_(2,2)_ = 0.90, *P* = 0.422) (Fig. 4). This suggests that transformation cues/triggers, if required, are not provided by the BL-like substitute Matrigel or the co-cultured cells. Thus, BL components such as laminin do not initiate transformation.

Transforming ookinetes often clump together and sink to the bottom of the culture wells, thus physical contact with other parasites or a solid surface may be required for transformation. This hypothesis was not upheld as parasites cultured in culture plates treated to become hydrophilic or non-treated plates and parasites maintained singly or at various densities still transformed into oocysts by day 2 of culture.

### 3.5 Is an extrinsic trigger required for transformation?

Following removal of both BL and co-cultured cells, we sought to determine whether ookinetes would transform into oocysts spontaneously, without any added trigger. PBS alone did not support transformation, therefore precluding time-dependent transformation in these minimal conditions. Viable ookinetes could be maintained in PBS for up to 7 days (e.g. viability on day 1, 84%; day 3, 65% and day 6, 5%). Removal of 100, 200 or 300 μl of the PBS and replacement with the same quantity of OCM stimulated transformation in a time and volume dependent manner (see Table 2). If still viable, ookinetes could be induced to transform up to 3 days post-PBS culture, thus suggesting that the transformation process will not be supported unless appropriate cues are received by the parasite, and that these cues are present in OCM in limiting amounts.

### 3.6 Minimal requirements for ookinete to oocyst transformation

The addition of single OCM components to PBS had varying effects on parasite development (Table 3). Notably, sodium bicarbonate (23.8 mM) alone supported transformation of ookinetes to live tooks, but parasites failed to complete the entire rounding-up process to form young oocysts. In contrast, a 0.2% lipid/cholesterol component was toxic to ookinetes, killing approximately 25% within 4 h of culture and all ookinetes within 24 h, and although a tenth of the concentration was not toxic, it did not stimulate transformation.

The addition of combinations of heat-inactivated FBS (HI FBS) and sodium bicarbonate or Schneider’s medium and sodium bicarbonate to PBS permitted complete transformation at maximal rates. However, the latter combination resulted in the appearance of a white precipitate, obscuring views of ookinetes and leading to the death of parasites within 2–3 days. Transformation also occurred when sodium bicarbonate was replaced with sodium carbonate, although a precipitate was again formed. Growth of viable oocysts proceeded for 5 days in PBS containing 15% HI FBS and 23.8 mM sodium bicarbonate (minimal media) The mean diameter of young oocysts increased from 7 to 8 μm on day 1 post-culture to 11–12 μm on day 5 (Table 4). However, maintaining the parasites in minimal media did not permit complete sporogonic development and oocysts were no longer viable after 7 days.

As serum is notoriously difficult to define, we substituted 15% FBS in PBS with 15% Albumax, but this failed to support transformation. We therefore tried to identify groups of components of Schneider’s medium (e.g. amino acids, salts, sugars or yeast) that alone might sustain development. None of these groups permitted oocyst development, however partial transformation did occur in the inorganic salts group (data not shown). These results indicate that, whilst bicarbonate alone may initiate transformation, complete transformation requires a combination of essential nutrients and thus transformation may be a two-stage process.

### 3.7 Media pH and transformation

As the pH of bicarbonate buffers is strongly influenced by pCO_2_ and hence sodium bicarbonate concentration, we adjusted the pH of OCM to establish if this alone was critical for transformation (Table 5). A high pH (9.0) greatly accelerated the rate of transformation; which began within 4 h of culture. However, at pH 9.0, GFP fluorescence was less intense and oocyst plasma membrane breakdown occurred from day 2 post-culture, after oocysts had attained diameters of 10–15 μm. Low pH (6.0) severely impaired oocyst growth. Transformation was maximal at pH 7 and 8.

## 4 Discussion

Using our in vitro system, we have been able to observe many thousands of ookinetes undergo transformation to young oocysts. Our observations of tooks in vivo have been far less frequent, as only a few of an invading population will be transforming at any one time. However, it is clear that this method of transformation certainly occurs in vivo. It would appear that the break-up of the inner surface membrane of the ookinete begins specifically along the convex side of the ookinete and spreads from this point, and there is likely to be an increase in the surface area of the single plasmalemma during this process. We were unable to detect remnants of the inner surface membrane in areas where a single plasmalemma was visible and the apical complex did not appear to undergo immediate de-differentiation (Canning and Sinden, 1973) but persisted until late into the took phase. The process of transformation thus appears to be similar to that of the sporozoite to the exoerythrocytic stage in the hepatocyte (Meis et al., 1983).

Ookinete to oocyst transformation represents one of the most severe bottlenecks for the malaria parasite, often reducing ookinete numbers to single digits (Dimopoulos et al., 2002). Culturing the mosquito stages of the rodent malaria parasite in vitro, in the absence of the unfavorable conditions generated in the blood meal bolus and by the insects’ innate defence system, permits transformation of the majority of live ookinetes to the oocyst stage and thereby suggests that most parasites have the potential to develop rather than undergoing apoptosis as they do in the midgut lumen (Al-Olayan et al., 2002b). The investigations reported here suggest that this transformation is not internally controlled but that specific external compounds are required to stimulate this process.

Some authors have made observations that suggest that the BL provides the necessary cues for oocyst transformation (Adini and Warburg, 1999; Arrighi et al., 2005). For example, when gametocytes are injected directly into the haemocoel, ectopic oocysts develop attached to the BL surrounding tissues such as the fat body and Malpighian tubules (Weathersby, 1954; Beaudoin et al., 1974). For these reasons, many culture protocols have employed a BL substitute (Matrigel) and it has been proposed that interactions between ookinete surface or microneme proteins and the mosquito BL play some role in oocyst development (for review see Siden-Kiamos and Louis, 2004). Here, we demonstrate conclusively that ookinetes will transform in the absence of any source of BL components such as laminin or collagen. Additionally, adhesion to a solid substrate or other cells is not required to trigger transformation. This may indicate that ookinete transformation is driven by soluble factors derived from the haemolymph.

Developing below the BL, in the subepithelial space, ookinetes and oocysts are bathed in haemolymph components, although direct contact with haemocytes is unlikely. Haemolymph from infected or non-infected *A. stephensi* mosquitoes has been recorded to have a pH ranging from 6.5 to 6.8 (Mack and Vanderberg, 1978). Whilst our in vitro investigation indicates that maximal initiation of ookinete to oocyst transformation and subsequent growth occurs within a pH range of 7–8 we did not examine events at pHs between 6 and 7, leaving the possibility that a pH in this range may be more favourable.

With the addition of sodium bicarbonate alone, ookinetes proceed to early stages of transformation as indicated by a change in shape, but do not complete development to young oocysts. It would appear that sodium bicarbonate is important for transformation and we suggest it may be a requirement for the first part of a two-step process. Following took formation, nutrients such as amino acids and sugars are needed for transformation to be completed and some growth to occur. These findings appear to reflect those reported in an investigation of triggers for in vitro exflagellation in *P. berghei*. Exflagellation could be stimulated in an extracellular bicarbonate concentration of 28 mM, whereas variation of the pH from 7.2 to 8.6 in a solution depleted of bicarbonate did not, alone, provide the stimulus (Claudianos et al., 2002).

The requirement for a range of nutrients for the second step of transformation suggests metabolite stores are severely limited in the ookinete and insufficient to sustain the immediate morphological changes that occur during complete transformation.

In contrast to the majority of parasites that infect mosquitoes, both *Hepatozoon caiman* (Lainson et al., 2003) and *Plasmodium* spp. develop underneath the BL on the midgut of their respective vectors, namely *Culex fatigans* and *Anopheles* spp., and do not enter the haemocoel immediately after exiting the gut lumen. This suggests that there may be an advantage to be gained by developing in this site. Recently, using double-stranded RNA (dsRNA) gene silencing technology, Arrighi et al. (2005) demonstrated that depletion of mosquito laminin led to a substantial decrease in oocyst numbers and suggested that the presence and integrity of the BL is vital for parasite development. So far, its role has not been elucidated but it may be of importance in vivo but not in vitro. The proposal that interactions between mosquito laminin and molecules in the oocyst capsule may result in the masking of the oocyst from the mosquito’s immune system, thereby protecting the developing oocyst from being melanised, has been recently supported by findings of Warburg et al. (2007). They demonstrated that beads coated in laminin or the ookinete surface protein P28 are protected from melanisation when injected into the mosquito haemocoel. It would be useful investigate this hypothesis further using a refractory, melanizing, strain of mosquito.

In conclusion, we believe we present the first description of an intermediary form between ookinete and oocyst, namely the took. Transformation of *P. berghei* ookinetes to young oocysts in vitro is not a simple time-dependent phenomenon; it requires a precise environmental stimulus, but not one provided by basal lamina components. The first stage of transformation is bicarbonate dependent, yet full transformation also requires nutrients and a suitable pH. Minimal requirements for transformation have been reduced in this culture system to 15% HI FBS and 23.8 mM sodium bicarbonate adjusted to pH 7.0.

These findings advance our understanding of the least studied phase of malaria parasites, that of the oocyst. They demonstrate the benefit that an in vitro system can bring to investigations of the biology of the mosquito stages of the parasite. In addition, re-evaluation of the potential for attenuated sporozoite-based malaria vaccines (Good, 2005; Waters et al., 2005) has brought to the forefront the need for competent systems to culture sporogonic stages of the life cycle.

## Figures and Tables

**Table 1:** Components of full-oocyst medium (OCM) per 100 ml

Oocyst medium component	Final concentration/100 ml
Sodium bicarbonate (mM)	23.8
Hypoxanthine (mM)	3.68
Penicillin (U)/streptomycin (mg)	10,000; 10
Gentamicin (mg)	20
*Para*-aminobenzoic acid (μM)	44
Foetal bovine serum (%)	15
Lipid/cholesterol (%)	0.2
Schneider’s medium (%)	85

Media were adjusted to pH 7 prior to filtration.

**Table 2:** Transformation of ookinetes to oocysts maintained in PBS

Oocyst medium added	Day 0	Day 1	Day 2	Day 3	Day 4	Day 5
400 μl added day 0	–	✓	✓✓	✓✓	✓✓	✓✓
						
100 μl added day 0	–	–	✓	✓✓	✓✓	✓✓
100 μl added day 1	–	–	–	✓	✓✓	✓✓
100 μl added day 2	–	–	–	–	✓	✓✓
						
200 μl added day 0	–	✓	✓✓	✓✓	✓✓	✓✓
200 μl added day 1	–	–	✓	✓✓	✓✓	✓✓
200 μl added day 2	–	–	–	✓	✓✓	✓✓
						
300 μl added day 0	–	✓	✓✓	✓✓	✓✓	✓✓
300 μl added day 1	–	–	✓	✓✓	✓✓	✓✓
300 μl added day 2	–	–	–	✓	✓✓	✓✓

Ookinetes were incubated in 400 μl of PBS which was then replaced with oocyst medium as indicated. A dose-dependent response was seen in the amount and speed of transformation. Data from ookinetes cultured in 400 μl of oocyst medium from the day of culture are displayed for comparison. Samples were taken from three independent experiments and media was replaced on day 3. –, no transformation; ✓, partial transformation (tooks); ✓✓, complete transformation of the majority of ookinetes to oocysts.

**Table 3:** Minimal requirements for triggering ookinete to oocyst transformation in PBS

Oocyst medium component	Day 0	Day 1	Day 2	Day 3
Full-media	–	✓	✓✓	✓✓
PBS alone	–	–	–	–
PBS + hypoxanthine	–	–	–	–
PBS + PABA	–	–	–	–
PBS + lipids	–	×	×	×
PBS + sodium bicarbonate	–	✓	✓	✓
PBS + Schneider’s medium	–	✓	✓	✓✓
PBS + HI FBS	–	✓	✓	✓✓
PBS + Schneider’s + sodium bicarbonate	–	✓	✓✓	✓✓
PBS + HI FBS + sodium bicarbonate	–	✓	✓✓	✓✓

Stages of transformation (compared with full-media) were assessed on days 0, 1, 2 and 3 post-culture when single and dual components of oocyst medium were added to PBS (see Table 1 for concentrations). Samples were observed from three independent experiments. –, no transformation; ✓, partial transformation (tooks); ✓✓, complete transformation to oocyst; ×, death of parasites. HI FBS, heat-inactivated FBS; PABA, *para*-aminobenzoic acid.

**Table 4:** Growth and viability of *Plasmodium berghei* oocysts over a 5 day period of culture in minimal media

Time post-culture	Day 1	Day 3	Day 5
Viability (*n* = 100) (%)	92	89	84
Mean oocyst size (*n* = 100) (μm)	7.80 ± 0.1	9.51 ± 0.17	11.36 ± 0.15
			
Example of oocyst			

Parasites were cultured in PBS containing 15% HI FBS and 23.8 mM sodium bicarbonate. One-hundred viable-oocyst diameters were measured each day, observed under phase contrast. Sizes are expressed as means ± SEM.

**Table 5:** Effects of pH on transformation rates

pH	Day 1	Day 2	Day 3	Day 4	Day 5
*Full-media*					
6	–	–	✓	✓	✓✓
7	✓	✓✓	✓✓	✓✓	✓✓
8	✓	✓✓	✓✓	✓✓	✓✓
9	✓✓	✓✓	✓✓	×	×
					
*PBS*					
6	–	–	–	–	–
7	–	–	–	–	–
8	–	–	–	–	–
9	–	–	–	–	–

Hepes (25 mM) was added to full-oocyst medium to retain the appropriate pH. PBS alone, irrespective of pH, does not permit ookinete to oocyst transformation. Full-oocyst media permits transformation at a wide pH range, but is inefficient or toxic at low and high pH’s, respectively. –, no transformation; ✓, partial morphological transformation; ✓✓, complete transformation to oocyst; ×, death of parasites.
